# And to end on a poetic note: Galen’s authorial strategies in the pharmacological books

**DOI:** 10.1016/j.shpsa.2011.12.019

**Published:** 2012-06

**Authors:** Laurence M.V. Totelin

**Affiliations:** School of History, Archaeology and Religion, Cardiff University, Humanities Building, Colum Drive, Cardiff CF10 3EU, UK

**Keywords:** Galen, Pharmacology, Compilation, Authority, Authorship, *peira*

## Abstract

This paper examines the authorial strategies deployed by Galen in his two main pharmacological treatises devoted to compound remedies: *Composition of Medicines according to Types* and *Composition of Medicines according to Places*. Some of Galen’s methods of self assertion (use of the first person; writing of prefaces) are conventional. Others have not received much attention from scholars. Thus, here, I examine Galen’s borrowing of his sources’ ‘I’; his use of the phrase ‘in these words’; and his recourse to Damocrates’ verse to conclude pharmacological books. I argue that Galen’s authorial persona is very different from that of the modern author as defined by Roland Barthes. Galen imitates and impersonates his pharmacological sources. This re-enactment becomes a way to gain experience (*peira*) of remedies and guarantees their efficacy.

## 1 Introduction^1^

The authorial strategies employed by Greek and Roman scientific writers have been the subject of various recent studies. Many of these studies have focused on grammar, and more particularly on the use of *ego*. Lloyd (1987, pp. 57–70) has examined the use of the first person in the earliest Greek scientific texts (those of the fifth and fourth centuries BC). von Staden (1994) has looked at the diverse uses of the authorial *ego* in Celsus’ *On Medicine* (1st century AD).^2^ van der Eijk (1997a) has examined various ‘rhetorical strategies’, including the use of the first and second person (pp. 115–119), deployed by Hippocratic writers and Aristotle (fifth and fourth centuries BC). Hine (2009) has attempted to assess the degree of subjectivity or objectivity of Latin technical and scientific works by studying the uses of *ego*, as well as those of first person plural pronouns and verbs, second person pronouns and verbs, and some impersonal verbal forms.^3^ Finally, Nutton (2009) has made a preliminary enquiry into Galen’s authorial voice (AD 129–216) by looking at his use of the first person and other grammatical modes of self assertion in the Latin translation of *De motibus dubiis*. Nutton concluded that Galen’s egocentrism led him to intervene as an author even when ‘there [was] no compulsion to do so’.^4^ Interestingly, these recent works do not engage—explicitly at least—with the literature produced in the wake of Foucault’s claims relating to authority and author-function in scientific texts before and after the eighteenth century (1969),^5^ and with Barthes’ assertion that the author is dead (1968). Neither do these studies settle on a definition of the words ‘author’ and ‘authorship’. In this paper, I will use Pamela Long’s definition:

On the most basic level, authorship refers to the act or practice of creating something, such as a treatise, a painting, or a material invention. In the most mundane sense, an author of a written work is a writer, who must always do something more than copy another text verbatim; copying two or more texts and putting them together may suffice.^6^

This definition is particularly useful for the texts studied here, Galen’s main pharmacological works, as they are compilatory in nature and often claim to copy material word-for-word.

Studying the grammar *stricto sensu* of an ancient technical text, however, is not the only way to assess its author’s degree of self-assertion. Thus, studies have recently been conducted into the forms, styles, genres, and organisational techniques (with a particular emphasis on prefaces) used by ancient scientific writers. The works of Asper (2007), König and Whitmarsh (2007), Fögen (2009), and Taub (2008; Taub & Doody, 2009) come to mind. Finally, several studies focusing on Galen have taken a more sociological approach to the physician’s methods of self-assertion. Here, some scholars have situated Galen in the context of the second- and third-century Roman Empire—an empire at its cultural, political and economic apogee.^7^ Others have looked more particularly at the context of the ‘Second Sophistic’, the first- to third-century movement that involved a revival of ‘classical’ oratory and was marked by an interest in the past and competitive displays of knowledge.^8^

In this paper, I wish to combine these approaches and examine Galen’s authorial strategies in his two main treatises devoted to compound remedies: *Composition of Medicines according to Types* (in seven books—abbreviated as ‘*Types*’ from now on) and *Composition of Medicines according to Places* (in ten books—abbreviated as *Places* from now on).^9^ These treatises are extremely long collection of remedies organised by methods of application in the case of *Types* (4 books on various plasters; 2 books on ‘multi-functional’, *polychresta*, drugs; and one book on emollient, laxative, and analgesic drugs) and in the ‘head to toe’ order in the case of *Places*.^10^ Thanks to cross-references, it can be established that Galen wrote *Types* before *Places*, and that he composed both treatises at the end of the second century AD or at the beginning of the third, and in any case after the great fire at Rome which destroyed many of his possessions in AD 192.^11^

Fabricius (1972) has shown that Galen relies mainly on a limited series of authorities in his collections of remedies: Andromachus (active in Rome in the 70s and 80s AD), Archigenes of Apamea (lived under Trajan), Asclepiades Junior, the pharmacologist (last quarter of the first century AD), Heracleides of Tarentum (Empiricist, 100–65 BC), Criton (lived under Trajan), and Heras of Cappadocia (practised in Rome between 20 BC and 20 AD).^12^ The pharmacological writers quoted by Galen, in turn, had consulted the works of hundreds of earlier medical authorities. The result is a massive (1686 pages in Kühn’s nineteenth-century edition) compilation of recipes produced in the Hellenistic and Early Imperial periods.

Galen’s pharmacological treatises are not his only compilatory works. Nor was Galen alone in producing compilations in the first centuries of the Roman Empire: many authors active around the time of Galen were engaged in similar activities. Thus, Pliny the Elder (AD 23/4-79, Latin), Plutarch (before AD 50—after AD 120, Greek), Aulus Gellius (born c. AD 125, Latin), Athenaeus (fl. AD 200, Greek), and Diogenes Laertius (3rd century AD?, Greek)—to name only a few—produced compilations of scientific, philosophical, literary, and biographical material.^13^ Like all these authors, Galen in the pharmacological treatises deployed various strategies to mark his stamp on borrowed material. One of Galen’s authorial strategies is, of course, to use the first person. However, looking at the use of the first person in a study of Galen’s authorial strategies is not sufficient, as authority can be expressed in a variety of other ways, which we will sample in the prefaces to *Types* and *Places*. We will see that these prefaces contain numerous tropes that are to be found in other Galenic writings and other ‘Second Sophistic’ texts. Whilst it is conventional to use the first person and to write prefaces to scientific texts, I will argue that Galen’s authorial strategies in the pharmacological books are original in two respects. First, he borrows the ‘I’ of his sources. In particular he borrows the ‘I’ of Damocrates, whose poems he uses to conclude several of his pharmacological books. Second, Galen often dwells on the derivative nature of his material by introducing borrowed recipes with the words *kata lexin* (in these words). I will reflect on the meaning of this expression, and conclude that the physician is doing more than plundering his pharmacological sources; he is taking on their voice, he is impersonating them.

## 2 The use of ‘I’

Galen sometimes uses first person (singular and plural) pronouns and possessive adjectives and very often first person verbs (again singular and plural) in his pharmacological treatises.^14^

From a grammatical point of view, pronouns are not compulsory in the Greek language; they are used to put emphasis on important points. Thus, the preface to *Places* contains two particularly interesting uses of the first person pronoun—one singular and one plural.^15^ Galen uses the phrase ‘those before us’ (*tois pro hēmōn*) to refer to physicians who have written remedies in the head to toe order in the past. With this use of the first person plural pronoun, Galen marks himself as a member of a community of learned physicians. The first person singular pronoun (*egō*) appears towards the end of the preface: ‘I, as Hippocrates ordered, have always attempted …’ Here, Galen is asserting himself very strongly, and declaring himself the true medical heir of Hippocrates—Galen is the ‘a new, less obscure, more accurate, more complete Hippocrates’.^16^

A noteworthy use of the first person plural pronoun is in the phrase *par*’*hēmin* (among us, at home) which Galen employs to refer to customs, plants, expressions from his home province of Asia. By means of this phrase, which appears 20 times in the pharmacological treatises, Galen presents himself as a Pergamene, often comparing the situation of his native city with that of Rome, as in the following example:

For there is an abundance of cold streams and snow in Rome, as there is too at home (*par*’*hēmin*) at Pergamum and in most of the cities of Asia and Greece.^17^

By the time Galen wrote the pharmacological books he had spent a lot of time in Rome, yet he still presented himself ethnically as a Pergamene, not as a Roman. In the words of Swain, he ‘insulated himself’ from the Roman world.^18^

The constant use of first person (singular and plural) verbs by Galen in the pharmacological treatises defies all classificatory attempts. I will limit myself to pointing out some common uses of first person verbs. First, Galen uses verbs in the first person as structuring markers, as signals to let his audience know what he is about to do, or what he has achieved.^19^ Second, Galen uses verbs in the first person to make claims to autopsy: he has observed something himself or he has used a remedy himself.^20^ This use of the first person is particularly important in a pharmacological compilation: it is plain that Galen could not have tried and tested all the remedies he transmits. Third, Galen uses verbs in the first person to say that he has changed (and improved) a recipe found in the works of his predecessors.^21^ These personal interventions in the first person allow Galen to make his own a collection of remedies he has plundered from various sources. However, one has to be careful as Galen sometimes retains first person verbal forms that were present in the sources he is excerpting. The ‘I’ of these sources becomes confused with Galen’s ‘I’.

Thus, Cajus Fabricius has noted that the verbal forms *chrōmai* and *chrōmetha* (‘I use’ and ‘we use’) occur more frequently in passages extracted from Andromachus than in those extracted from Asclepiades.^22^ Fabricius inferred from this observation that Galen was truly replicating his sources verbatim, copying even their use of the first person. Other examples of Galen’s replicating the first person of his sources are easy to find. For instance, Galen repeats twice in *Types* the recipe of a ‘Lucius, our teacher’ (*ho hēmeteros kathēgētēs*).^23^ This teacher, however, is not that of Galen, but that of Asclepiades, whom Galen is excerpting. Galen is here quoting Asclepiades verbatim, although the reader has to go back several pages in the modern edition to find the claim that Galen is quoting *kata lexin*.^24^ It is also interesting to note that Lucius’ recipe is written in the first person plural. Either Aslepiades or his source must have cast this recipe in this particular grammatical format. The ‘we’ here is not Galen’s ‘we’; the physician is simply copying his source.

Writing recipes in the first person plural seems to have been a style favoured by Asclepiades. Twice in *Places* Galen gives a recipe of a golden remedy excerpted from Asclepiades, and cast in the first person plural:

Straight after [the previous recipe], Asclepiades wrote another thus. Emollient which **we call** (*kaloumen*) golden: golden arsenic and asbestos, of each 2 ounces; moist alum, 2 ounces; split alum, 2 ounces; **we chop** (*koptomen*) the dry ingredients, **we sieve** (*sēthomen*) them, putting them in a mortar; to these **we add** (*epiballomen*) 8 kyathoi of vinegar, grinding carefully as we mix with (*analambanomen*) the melted ingredients. These are wax, torch and pine resin, of each 2 litrai; common oil, 8 kyathoi. Melting these, **we leave** them (*eōmen*) to cool down, and scraping them **we add** (*epiballomen*) them to the ground ingredients.^25^

So far, it is clear that the ‘we’ is Asclepiades’ ‘we’. But what about the next sentence, also written in the first person plural and starting with a strong first person pronoun?

**We** (*hēmeis*) **have added** (*prostetheikamen*) to the preparation of the remedy 2 ounces of myrrh, and others (*heteroi*) have added 2 ounces of realgar, removing the 2 ounces of moist alum. And [now] **we** (*hēmeis*) **prepare** (*skeuazomen*) it by including all the aforementioned ingredients, and it becomes more efficacious.^26^

Are these comments still by Asclepiades, or are we now reading the comments of Galen on how to improve the remedy? Is Galen intervening in the first person plural after reproducing a recipe written in the same grammatical form? These questions are almost impossible to answer, unfortunately.

Examples of borrowing of the first person in the pharmacological writings could be multiplied (we will see two further examples later), and it is interesting to note that similar cases of borrowing have been observed in collections of Galen’s clinical case histories.^27^ Of course Galen does acknowledge his borrowings on most occasions, but one often has to read back several dozens of lines to find the claim to verbatim quotations. One should also stress that, in a system of writing where there was relatively little use of punctuation, such as that used at the time of Galen, it might have been quite difficult at times to determine where Galen’s quoting ended and his own comments in the first person started.^28^ From the point of view of a modern reader, highly concerned with intellectual property, this borrowing of the ‘I’ seems rather alien.^29^ However, in an ancient pharmacological tradition, based on experience (*peira*), this practice makes sense, as argued by Armelle Debru in a study of Aetius’ borrowing of Galen’s ‘I’ (1992). In the Empiricists’ tradition, experience (*peira*) is the product of three factors (the empiricist tripod): *empeiria* (personal experience); *historia* (data provided by others); and transition by way of similars (where use of analogy directs transition of knowledge).

Galen himself was not an Empiricist, but he did stress on multiple occasions that, in the field of pharmacology, one particular type of experience, qualified experience (*diōrismenē peira*), takes precedent over reasoning in acquiring pharmacological knowledge.^30^ He also quoted, sometimes first hand, sometimes through his sources, the recipes of many Empiricists. In an empiricist, or empiricist-inspired framework, borrowing recipes is a form of *historia*: the compiler collects data provided by tradition. Galen does on several occasions stress the importance of *historia* in pharmacology. For instance, he writes in *Places* 1 that ‘the confidence in remedies is increased through concordant *historia*, and for that reason I write all the remedies [against alopecia] from the Empiricist physicians’.^31^ Of course, a careful medical compiler should only select recipes whose principles he agrees with (or signal his disagreement)—recipes he could have concocted himself. In this context, the borrowing of the ‘I’ becomes more understandable: the compiler preserves the ‘I’ not only for the sake of verbatim quotation, but also because he feels he could have made these observations himself. In other words, in an empiricist model of knowledge, the individual ‘I’ and the ‘I’ of tradition (the collective ‘I’) can become very blurred indeed.

It is also worth reflecting on the notion of ‘verbatim’ quotation. As already mentioned, Galen often acknowledges the fact that he copies passages word for word, marking his borrowing with phrases such as *kata lexin* (in these words), *outōs* (thus), and *ōde pōs* (in this way). ‘*Kata lexin*’, he uses particularly frequently in the treatises under consideration: 109 occurrences of this phrase are to be found in these pharmacological treatises, for 172 occurrences in the Galenic corpus, and 356 in the corpus of Greek texts (the *Thesaurus Linguae Graecae*) up to and including the third century AD. Since Galen commented on literary forgeries on several occasions, one might think that, when using the phrase *kata lexin*, he is attempting to avoid plagiarising his pharmacological predecessors.^32^ I would argue, however, that avoiding plagiarism is here less important to Galen than calling on the authority of his pharmacological predecessors. Let us think about the meaning of the phrase *kata lexin*. *Lexis* refers to oral utterances as well as to written words. When Galen uses the phrase, he makes his sources talk, quite literally. He calls his sources to witness and makes them testify to the efficacy of the remedies he is transmitting; again it is *peira* that is the centre of Galen’s pharmacological work. However, most of the authorities who are ‘made to talk’ in the pharmacological books are long dead, and Galen must impersonate them. He takes on the voice (*lexis*) of his sources and utters their words almost as if an actor. This idea of Galen as the actor, I will develop further at the end of this article.

To sum up, Galen uses the first person on a regular basis in the pharmacological treatises. However, his ‘I’ is not always a modern individual ‘I’, it is an ‘I’ that is the product of a tradition, where *peira* (expertise) is accumulated through centuries of pharmacological observations. The act of compiling is in itself a method of research. Studying the use of the first person is useful, but it can lead to confusions. It can also lead to omissions, as Galen could establish his authority without using the first person. A good place to sample this is in the prefaces to *Types* and *Places*, where Galen asserts his authority without always using the first person. These prefaces will also allow us to ask which type of authority Galen wishes to establish.

## 3 Galen’s construction of authority in the prefaces to the pharmacological works

Prefaces to ancient technical texts have recently attracted the attention of scholars, since, in collections of borrowed material, they are the place where the compiler can establish his authority.^33^ These studies have shown that prefaces to ancient scientific works are often rather conventional and rely on a stock of tropes. As we will see, Galen’s prefaces to the pharmacological treatises are no exception and make use of numerous *topoi* common to this type of texts.

In the preface to *Types*, Galen starts with an autobiographical anecdote, telling his reader that he had already written two books on remedies classified by types, but that they had been destroyed in the great fire of 192, without any of his friends having copies (points 1–2).^34^ It is at the pressing request of his friends that he writes again (point 3). He explains what the lost books contained, using this opportunity to launch an invective against various categories of physicians (which are not mutually exclusive): the physicians who do not give the *epangelia* of a recipe, that is, the part of the recipe where the properties of the preparation are explained (points 4–6); those who think that in the mixing process opposite powers of drugs cancel each other (points 7–10); and the Empiricists who find remedies by chance (point 11). In the context of the invective against the physicians who do not give the *epangelia*, Galen uses a quotation from an unknown tragedy as a means to laugh away a serious matter;^35^ and in the second invective, he mentions his treatise on simple remedies. Galen only abandons his invective in the final sentence of the preface (point 12), where he refers to his aim as *gymnasia*, training. Earlier (point 10), he had referred to his pharmacological writings as ‘useful teaching’ (*chrēsimon didaskalian*). The use of first person pronouns is quite prominent throughout this preface to *Types* (7 occurrences); that of first person verbs slightly less so (4 occurrences).

In the preface to *Places* Galen starts by reminding the reader of his works where he exposed the method of compounding remedies: *Method of Healing* and *Types* (point 1). He then justifies his reason for writing remedies in the head to toe order (point 2). This is followed by a passage on the generation and diseases of the hair, with a mention of yet another one of his works, *Mixtures* (points 3–4). In the final paragraph (points 5–7), Galen criticises those doctors who apply remedies without method (*chōris methodou*) and invokes the Hippocratic principle whereby one should ‘help and at least cause no damage’. Galen tells his reader (addressed in the second person) that he will be able to learn (*mathein*) this method by studying his works. The first person is not used as much as in the preface to *Types* (1 first person verb; 3 first person pronouns), but, as pointed out earlier, it is employed in a very dramatic fashion to establish a parallel between Galen’s method and that of Hippocrates, the father of medicine. Even though most of this preface is in an impersonal style, the personality of Galen is apparent throughout.

Whilst they are presented differently, these two prefaces convey similar messages. First, Galen is reluctant to write and has to justify himself. This motif, which is to be found in many works written in the first centuries of the Roman Empire, is particularly visible in the preface to *Types* but also present in that to *Places* (where Galen justifies his reasons for writing yet another treatise on compound remedies).^36^ Galen is justified in writing because he is teaching his audience. In the preface to *Places*, this audience is referred to in the second person singular and mention is made of learning; in the preface to *Types*, Galen addresses his friends (*philoi*) and companions (*hetairoi*), that is, his learned students.^37^ Throughout the pharmacological treatises, Galen sometimes interrupts the flow of recipes to address his audience in the second person and refers to their learning and his teaching.^38^ Addressing friends is also a trope that frequently recurs in the writings of Galen and his contemporaries.^39^

Galen’s pharmacological teaching will be useful (*chrēsimon*). Again, the notion of utility is a motif in ancient scientific prefaces.^40^ What Galen is trying to teach is the best method to compound remedies. The word *methodos* and cognates occur very frequently in the pharmacological treatises (109 times for 862 in the Galenic Corpus).^41^ It is not explicitly said in either preface what this method is exactly, but more information can be found at *Types* 2.1:

It is possible for those who have been trained (*gumnastheisin*), as I say, beyond being capable to compound useful (*chrēsima*) drugs, to be able to judge the remedies prescribed by the physicians before us, and to use all their remedies in a suitable manner, and to find remedies by following the method that has been taught (*didachthēsomenēn methodon*).^42^

Galen’s method gives shape and originality to his pharmacological compilation.^43^ It has been developed through years of experience, and the writing of several works on therapeutics, which Galen mentions several times in his pharmacological books: *Method of Healing*, a work in 14 books, whose third book is devoted to the theory of compounding remedies but gives few examples; *Powers and Foculties of Simples* in 11 books, whose knowledge is essential to the compounding of remedies; and *Mixtures* in 3 books, where he exposes the principles of his humoural theory.^44^ Ultimately, through the correct use of Galen’s method, his student will be able ‘to help and at least not to cause harm’—Galen thus links his method to the famous Hippocratic principle, expounded in *Epidemics* 1.5.^45^

Galen is adamant his method is better than that of most of his pharmacological predecessors, who wrote ‘without method’ or ‘without distinction’.^46^ Against these faulty predecessors, Galen uses invective, both in the prefaces and in the main body of his pharmacological books. Through invective against his predecessors, and more positively, through his appropriation of the Hippocratic tradition, Galen is, to use Heinrich von Staden’s expression, ‘staging the past’. This staging of course has for ultimate aim to place Galen in a good light: ‘The public crafting of the self … often involves the staging of others, and especially of one’s difference from or similarity to others.’^47^ In his use of invective and his staging of the past, Galen is similar to other participants in the Second Sophistic, who were constantly referring to the classical past.^48^

The object of Galen’s pharmacological invectives is sometimes named, as in the following passage, which allows us to determine that one of the invectives in the preface to *Types* was directed at Andromachus:

Andromachus collected many remedies … He included them in three books … One may blame him, as I said, for specifying neither the method of preparation (*skeuasia*), nor the places of application, nor the powers, nor the *epangelia*.^49^

More often, however, Galen criticises all members of a particular medical sect (as the Empiricists in the preface to *Types*, or the ‘so-called Methodists’, who are mentioned on several occasions in the pharmacological treatises).^50^ And most often, Galen lumps those pharmacologists he condemns under vague designations such as ‘most of the physicians’ (the *hoi polloi tōn iatrōn* mentioned in the preface to *Places,* point 5), or ‘the most recent [physicians]’ (*hoi neōteroi*).^51^

One recent authority, however, is completely immune to criticism, and is even praised on several occasions by Galen: Damocrates. In the final section of my paper, I will discuss Galen’s attitude towards Damocrates and note that his poems are sometimes used to conclude pharmacological books. This use of Damocrates’ poems as conclusion was, I will argue, a deliberate strategy on the part of Galen.

### 3.1 A poetic concluding note

Servilius Damocrates, freedman of M. Servilius, was active under Nero and Vespasian.^52^ He wrote recipes in iambic trimeters, which Galen praises three times in *Types* and twice in the treatise on *Antidotes*.^53^ Verse recipes are best for two reasons: first they are more accurate (they help preserve the proportions of the drugs better) and second they are easier to remember.^54^ This makes them particularly useful in the context of teaching, which is the aim of Galen’s pharmacological books.^55^ Not all verse recipes, however, fit this bill. For instance, the verse recipe (elegiac couplets) for Theriac by Andromachus the Elder, which Galen transmits in *Antidotes*, is so unclear that it warrants a rendition into prose (by Andromachus the Younger) and into ‘simpler’ verse (by Damocrates).^56^ According to modern standards, the poetry of Andromachus is more beautiful, and for that reason it has been studied more, but it is Damocrates’ plain Iambic poetry that appealed to Galen.^57^ In fact, Galen’s attitude towards poetry was rather ambivalent. Poetry, in Galen’s opinion, could convey untruths.^58^ We have seen that, at the beginning of *Types* 1, Galen introduced an untruth with a quote from tragedy. It was used in jest as part of a criticism of pharmacologists who, like Andromachus, do not specify the *epangelia* of a remedy. What Galen disliked most about poetry was its constant recourse to metaphors; he had nothing against verse per se.^59^ Quite the contrary, Galen wrote several books on comedies (now lost), which were devoted to the ‘ordinary words’ of comedy (*politikōn onamatōn*).^60^ Galen tends to appreciate poetry less for its beauty than for its utility: Damocrates’ poetry is more useful for didactic purposes than that of Andromachus and therefore more praiseworthy.^61^

One of the uses Galen finds for poetry is as a conclusion for his pharmacological works. He concludes five of his pharmacological books with one or several verse recipe by Damocrates: *Places* 5 (three verse recipes);^62^ *Places* 8 (four verse recipes); *Types* 1 (one verse recipe); *Types* 6 (five verse recipes); *Types* 7 (final book, four verse recipes). By concluding several of his pharmacological books with verse recipes, Galen implicitly reiterates some of the principles that should define the good pharmacologist: he is acutely aware of the need for accuracy; he has culture; and he wants to teach simple ways to remember pharmacological knowledge. This use of verse recipes to conclude pharmacological books—and in the case of *Types*, the entire treatise—comes quite close to a ‘conclusion’. Galen coated the rim of his bitter pharmacological treatises with poetry.^63^ In Greek technical literature, where conclusions are generally absent, this is worth stressing.

But there is more. Damocrates’ poems which conclude *Types* 6 and *Types* 7 both end with statements in the first person singular. The final three lines of the poem closing *Types* 6 are a first person intervention:

**I** (*egō*) **took** away the chalk and **added** (*eballon*) twice the amount of alum and **I knew** (*egnōn*) that the remedy worked better, as it caused no blisters.^64^

The author of this intervention explains how he modified (and improved) a recipe against perspiration. No doubt Galen would have been able to compose a few iambic trimeters, but he does not usually express himself in verse. The ‘I’ of the closing sentence of one of his pharmacological books therefore appears not to be Galen’s but Damocrates’ ‘I’.

The final lines of the poems concluding *Types* 7, and therefore the final lines of the entire treatise, are also an authorial statement:

**I noticed** (*epegnōn*) that some use much suet, much marrow, some aromatic substances, juices, and some other perfumes. And, on the contrary, having used them, **I knew** (*egnōn*) that such mixtures are vulgar and undistinguished, but that their nature is simple.^65^

Whether Galen is here replicating the verses of Damocrates or copying his style to intervene in the first person, it remains that *Types* 7, and therefore the entire treatise *Types*, ends on a personal statement cast in poetic form. This is a particularly strong way of closing a treatise.

As Galen affirms on several occasions, poetry facilitates the act of memory. This is most probably because verse lends itself to oral performance, and one may assume Damocrates’ pharmacological poems were ‘performed’ by Galen in front of his learning audience. Much of the recent literature on the scientific works produced in the context of the Second Sophistic has stressed the importance of display (*epideixis*) and staging. Thus Galen’s public dissections and vivisections have been compared by von Staden to oratory performances and by Maud Gleason to both public criminal interrogations and arena spectacles.^66^ Galen’s pharmacological activity may have been more private than his public dissections; they do not appear to have involved large crowds.^67^ However, Galen’s pharmacology may have also had some element of theatricality in that the physician sometimes took on the mask and the voice (*lexis*) of his pharmacological predecessors; he impersonated them to lend authority to the recipes he was transmitting. To the modern reader this might seem rather strange, but Galen lived at a time when, as noted by Gleason, orators impersonated statues, funerary epigrams addressed passers-by and objects were made to stand trials.^68^ In her study of the orator Favorinus (c. AD 85–155; a congenital eunuch), she writes:

To assume and then assimilate personae was to become oneself. It is this sense that we can apply to Favorinus a paradox coined by the arch-poseur of another age: ‘Man is least himself’, wrote Oscar Wilde, ‘when he talks in his own person. Give him a mask and he will tell you the truth’.^69^

Galen, of course, did not face the same identity issues as Favorinus. However, like Favorinus and like all technical authors of the time, he was concerned with constructing his own authority in the pharmacological books. Borrowing his predecessors’ ‘I’, impersonating their recipes *kata lexin*, concluding some of his pharmacological works with Damocrates’ poems—all forms of impersonation—helped him to build that authority.

## 4 Conclusion

Galen’s pharmacological works are derivative and compilatory in nature. Yet the Pergamene establishes his authority throughout these works. He does this in a variety of ways in his prefaces and throughout the treatises: he uses the first person; he boasts about his method and his experience; he stages his predecessors to appear in the best light possible. This authority Galen sometimes establishes in the first person, sometimes not. In any case, one should always be careful not to have too modernising a view of Galen’s role as an individual author. Galen certainly is not the author defined by Barthes, that author who ‘closes writing’, but he is an author in the limited sense given by Pamela Long. His role, like that of many compilers, is closer to Barthes’ notion of a reader. In the words of Max Thomas who discussed the compiler’s role in Renaissance commonplace books:

The compiler, then, operates in ways similar to Barthes’ notion of a reader: ‘someone who holds collected into one and the same field all of the traces from which writing is constituted’: not as someone who acts as a terminus; rather someone who channels the energies of poetic discourse and then reintroduces them into the cultural flow from when they were written/read.^70^

In the case of Galen, the notion of ‘reader’ can be taken, I have suggested, quite literally. Galen reads out loud (or has read to him), keeps in memory, and performs back the recipes he has received from tradition. Galen stages his predecessors, he re-enacts their recipes *kata lexin*. He calls on their experience to guarantee the efficacy of the remedies he is transmitting, and in the process, he creates his own persona: a mimetic, proteiform, theatrical ‘I’.

## Appendix A Preface to *Types* 1 (K13.362–367)

1. I had already written a study [on this topic], having published its two first books and left them with other [books] in the storehouse by the Via Sacra at the time when the entire Temple of Peace and the large libraries by the Palatine were destroyed by fire.
2. For at that time, my books and those of many others, which had been deposited in that storehouse, were destroyed, since none of my friends in Rome had agreed to have copies of the two first [books of my study].
3. Thus, as my friends pressed me to write immediately the same study, it seemed necessary to me to say a word of the previous editions, lest someone who encounters them wonders why I have written twice on the same topic.
4. In these works, from the beginning of the first book, I argued as much as possible against those who use sophistry to remove the *epangelia* of compound remedies, and in some cases even disparage [the *epangelia*] of simple drugs, and at the same time ridicule those who have written as title for these drugs that they are helpful for the spleen, or the kidneys, or the bladder, or the head, or the lungs.
5. For is it the case, they say, either that someone gives them order, as in tragedy: ‘Go, you, to the pomegranate trees of Inachos, and march around Cadmeian Thebes’, thence, in that way, enter the gates to the liver, and to the bladder or the two kidneys; or is it the case that what is swallowed into the belly is delivered to the liver, and thence carried to the entire body?
6. But this jest shows that those who say this are so completely inexperienced in the power of drugs that they ignore that, of all the organs of the body, the lung only is ulcerated by the sea-hare, and that again only the bladder [is ulcerated] by cantharid beetles … [more examples]
7. I have brought to completion this argument in a variety of ways in the first of the treatises which I had already written, as I said, and likewise, in the second book [of that lost work], I worked out another argument, since some [other people] think that in the mixing process the opposite powers of simple drugs cancel each other completely.
8. Thus if, they say, you mix the hottest water with the coldest, neither of the two mixed substances will remain as before, but some third substance will be generated, different from both.
9. These have been shown to talk nonsense because they do not realise that some of the drugs that are applied to the body act by means of innate powers, while others act by means of acquired qualities … [various examples of qualities]
10. Thus, having determined all these [qualities] in the treatises on the power of simple drugs, which are eleven in number, I will now turn to the useful teaching, in which someone has been trained whenever, even if he is not given complete recipes of polypharmaceutical remedies, he is not at a loss as to their utility against each affliction, but if he is, as I said, experienced in the matter of simple drugs.
11. In addition this argument shows the contentiousness of the Empiricists who refer almost all compound drugs to dreams, chance and accident, except for a very few drugs, which they say, have been discovered in accordance with the common reasoning of all men, which they themselves call *epilogismos* … [examples]
12. It is now time to talk about the particular training, using examples for each disease.

## Appendix B Preface to *Places* 1 (K12.378–381)

1. It has been shown earlier in the works *on the Method of Healing* that it is not only the disease to be treated that indicates the cure, but that the *krasis* of the sick and the nature of the suffering part also indicate the treatment in no small way, as indeed I have shown the entire method whereby one might make compound remedies from simple remedies in short in these books [*Method of Healing*], and more at length in the seven books before these [i.e. *Types*].
2. Now in order for someone to prepare [remedies] himself and use correctly this method and the discoveries of the previous doctors, it has seemed good to expose the properties of the remedies according to the parts of the body, starting with the head, like all the doctors before us.
3. More common diseases occur to the head than to any other part, but afflictions of the hairs have little in common with other diseases; and for that reason, few [authors] start their discourse with the generation of the hair.
4. For the growth of the hair is of the same kind as that of the plants growing out of the earth, as has been shown in the books *On Mixtures* … [passage on the generation and diseases of the hair with one occurrence of the first person plural].
5. And when they use the remedies which they have themselves written, many doctors not only act by chance, sometimes very much so, but they also cause a lot of damage, so that they make the condition incurable, in the same way as what happens to those who use these and many other remedies without method.
6. As Hippocrates ordered, I have always attempted to act in the works of the art in order to offer help, as he has written: ‘to help or at least not to harm’.
7. It will be possible for you to learn thence that the use of remedies without method not only never helps but often causes harm. For the time to start the discussion itself has come.

## Footnotes

1 All references to Galen’s texts given here include a reference to their modern editor. All references to Kühn (1821–1833) are abbreviated as in the following example: K12.535.4, where K = Kühn; 12 = volume number; 535 = page number; 4 = line number.

2 von Staden finds five different uses of *ego* in Celsus: (1) nomenclative *ego*; (2) *ego* of disposition; (3) autoptic or empirical *ego*; (4) *ego* as reader; (5) *ego* of scientific independence.

3 Hine classifies the uses of *ego* in the following manner: (1) uses of *ego* relating to the author’s relationship to dedicatee; (2) uses of *ego* relating to experience; (3) uses of *ego* relating to the author’s skills; (4) uses of *ego* relating to the author’s judgement; (5) uses of *ego* relating to the author’s skill as an instructor.

4 Nutton (2009, p. 61). *De motibus dubiis* survives only in Latin translation, but the translation by Niccolò da Reggio is so literal that it can be used to study Galen’s use of the authorial *ego*.

5 Foucault (1994) [1969], especially pp. 799–800; see Chartier (2003).

6 Long (2001, pp. 7–8).

7 See in particular Flemming (2007).

8 See Bowersock (1969, pp. 59–75), Reardon (1971, pp. 45–63), Kollesch (1981), Pearcy (1993), Swain (1996, pp. 357–379), von Staden (1995a, 1997a), Mattern (2008, pp. 7–11); articles edited by Gill, Whitmarsh, & Wilkins (2009).

9 The latest edition of these texts is that by Kühn (1821–1833), which is unfortunately deficient in many ways. Galen composed several other pharmacological treatises: *Powers and Faculties of Simples* (in eleven books), *Antidotes* (in two books), and *Theriac**to Piso*. The treatise *Theriac**to Pamphilianus* is not authentically Galenic, but dates to the time of Galen (see Nutton, 1997). For an introduction to Galen’s pharmacology, see Vogt (2009).

10 On the organisation of these treatises, see Fabricius (1972, pp. 19–23) and Vogt (2009, p. 311). On organisation principles in Galen more generally, see Flemming (2007).

11 See Ilberg (1896, p. 195), corrected by Fabricius (1972, pp. 23–24). The fire is mentioned in the preface to *Types* 1 (K13.362), see below for more detail.

12 For information on these pharmacologists, see Fabricius (1972); relevant articles in Keyser & Irby-Massie (2008).

13 The ancients would not, however, have used the word *compilatio* to refer to their activity, as it had negative connotations. See Hathaway (1989) on the story of the word *compilatio*.

14 Same observation in Nutton’s study of *De motibus dubiis* (2009, p. 58).

15 K12.379.5 and K12.381.4. The preface contains a further use of the first person plural pronoun.

16 von Staden (2009, p. 134). On the position of Hippocrates in Galen’s pharmacological works, see Fabricius (1972, pp. 203–205) and Jouanna & Boudon (1997). See also below for more detail.

17 *Places* 2.1 (K12.508.7–10). On this expression, see Schöne (1917, pp. 107–109) (incomplete list, which can be completed by searching the phrase in the *Thesaurus Linguae Graecae.* In total, there are 129 uses of this phrase in the Galenic Corpus), Nutton (1991, p. 23).

18 Swain (1996, pp. 377–378). See also Nutton (1991, p. 23, 1995, pp. 365–366), Flemming (2007, p. 246), and Mattern (2008, p. 49).

19 See for instance *Places* 1.2 (K12.383.5-8): ‘After the purgation of the entire body, it is best to purge away the phlegm, and then to use local remedies, whose composition I set out (*erchomai*) to describe.’

20 Galen’s own observations: see for instance *Places* 3.1 (K12.600.7-11): ‘Concerning earaches caused by cold. Heating remedies cure very fast the pains caused by the cold only. And I saw (*oida*) some rustics scoop out some large onions, then fill them with oil and heat them lightly in ashes, then pour it in the ears’. Galen’s use of a remedy: see for instance *Places* 6.7 (K12.956.11-13): ‘[Remedy] of Soranus. We know through experience (*pepeirametha*) that the remedies prescribed by Soranus work well, which he wrote thus.’

21 See for instance *Places* 1.2 (K12.403-404): ‘Concerning [the recipes] prescribed by Cleopatra in the *Cosmetics*. In Cleopatra’s *Comestics,* remedies against alopecia are prescribed, in her very own words, as follows. Against alopecia: pound fine realgar, and mix it to mix it to oak-coppice. Having cleaned [the head] as much as possible with soda beforehand, stuff [the preparation] in a cloth and apply. I (*egō*) added (*prosemixa*) *aphronitron* to the above mentioned [ingredients] and it worked well.’

22 Fabricius (1972, pp. 31 and 174–179).

23 *Types* 2.17 (K13.539.4-7) and 3.9 (K13.648.12-15): ‘[The recipe] which our teacher Lucius used is as follows: first we melt (*tēkomen*) suet and oil, and when it is dissolved, we sprinkle on (*katapassomen*) fine frankincense powder and litharge.’

24 The claim is at *Types* 2.17 (K13.535.12-14): ‘Concerning the plasters for fractures [preserved by] Asclepiades. He [sc. Asclepiades] wrote the first [plaster] in these words in the third book of external remedies.’

25 *Places* 10.2 (K13.341-342) and 9.2 (K13.251) (with some minor differences). This recipe is mentioned (but for another purpose) by Fabricius (1972, p. 115).

26 These comments are shorter at *Places* 9.2 (K13.251), they end after ‘2 ounces of myrrh’.

27 See Mattern (2008, p. 38).

28 See Mattern (2008, p. 217 note 123). On ‘separators’ used in recipe collections and other medical texts, see Hanson (1997, p. 302).

29 Long (2001, pp. 10–12) draws a distinction between ‘intellectual property’, which was not a concern in antiquity, and plagiarism/theft, which was a concern. On plagiarism in antiquity, see also Silk (1996).

30 See van der Eijk (1997b), and in particular p. 36 for references. On medical experience in antiquity, see von Staden (1975) and Grmek & Gourevitch (1985).

31 *Places* 1.1 (K12.410.4-7). See Fabricius (1972, pp. 48–50) and Jacques (1997, pp. 117 and 118), where other mentions of *historia* in Galen’s pharmaceutical treatises are discussed.

32 Galen, *On my own Books*, prooemium (134 Boudon-Millot, 2007b), *Commentary to Hippocrates ‘On the Nature of Man’* 1.44 and 2.1 (55.6-14 and 57.1-21 Mewaldt et al., 1914). See Hanson (1998, p. 33).

33 See for instance Janson (1964) on Latin prose prefaces in general; the works edited by Santini & Scivoletto (1990–1992) on the prefaces to Latin scientific texts; Formisano (2001, pp. 27–31) on prefaces to Late-Antique scientific texts; König (2009).

34 This fire is mentioned in *On my own Books* 3.7 and 3.14 (143–144 Boudon-Millot, 2007b), *On the Avoidance of Grief* is devoted to the losses Galen suffered in the fire; the text’s *edition princeps* is by Boudon-Millot (2007a). Translations of the prefaces to *Types* and *Places* can be found in appendix to this article.

35 Snell & Kannicht (1981)*Adespota* 177.

36 Galen also expresses reluctance to write in *On the Order of my Books* 1.5 (89 Boudon-Millot, 2007b). See van Gröningen (1963, pp. 2–3) and König (2009, pp. 56–57). See König (2009) for references to further examples of this motif.

37 On these friends and companions of Galen, see Jacques (1997, pp. 104 and 121), Hanson (1998, p. 25), and Mattern (2008, pp. 25 and 211, note 66). For the close equivalence of *philos* and *hetairos* in this period, see Konstan (1997, p. 121).

38 See for instance *Places* 8.1 (K13.124.9-12): ‘These things have already been said sufficiently in the works *on the Method of Healing*, and it is clear that, to those who have been trained (*gegumnasmenois*) by means of these books, the teaching (*didaskalia*) which we have now started is useful (*chrēsimos*).’ On the use of *didaskō* (to teach) and related words by Galen, see López-Férez (2003).

39 See König (2009) for examples.

40 See Formisano (2001, p. 29) for examples. In the writings under consideration, the notion of usefulness is often related to those of precision (*akribeia*) and clearness (*saphēneia*), on which I will come back later.

41 See Jacques (1997, p. 107). Of these occurrences, some refer to the Methodists, whom Galen calls ‘the so-called Methodists’ (see below for references) and some refer to *Method of Healing*.

42 *Types* 2.1 (K13.459.6-11). On Galen’s method in the pharmacological books, see Jacques (1997), van der Eijk (1997b), and Vogt (2009). On Galen’s *methodos* more generally, see Boudon-Millot (2009).

43 See König & Whitmarsh (2007, p. 9) on the possibility of originality in compilations.

44 See Jacques (1997, p. 123).

45 See Jouanna & Boudon (1997).

46 On pharmacologists failing in the eyes of Galen, see von Staden (1997b).

47 von Staden (2009, p. 132). On historical awareness in ancient medical writing, see van der Eijk (1999).

48 See von Staden (1997a, p. 52).

49 *Types* 1.16 (K13.441.12–442.2). Galen also gives histories of pharmacology at *Types* 2.5 (K13.502), 3.9 (K13.642), *Places* 6.9 (K12.989), 7.1 (K13.13). See Fabricius (1972, p. 43) and Jacques (1997, p. 121).

50 *Places* 2.1 (K12.513.13), 2.2 (K12.571.13), 8.1 (K13.117.13).

51 See von Staden (1997b, pp. 65–66).

52 On Damocrates, see Bowie (1997), von Staden (1998) and Vogt (2008). His poems are edited by Bussemaker (1851, pp. 99–132).

53 *Types* 1.19 (K13.455.6-10): ‘Damocrates on the white plaster. Since the powers of drugs written in verse are easiest to remember (*eumnēmoneutoi*) and preserve accurately (*akribē*) their proportions, it has seemed best to me to mention …’ *Types* 5.10 (K13.820.14-17): ‘The remedy with dittany according to Damocrates. I have often said that verse recipes are more useful (*chrēsimōteroi*) than prose recipes, not only for the sake of memory (*eis mnēmēn*), but also for the precision (*akribeian*) in the proportions of the various ingredients.’ *Types* 7.8 (K13.988.4-7): ‘Emollient of Damocrates. Since verse recipes are useful (*chrēsima*) for memory (*pros mnēmēn*) and maintain exactly (*akribōs*) the proportions of the remedies, for this reason, I have taken the habit of listing the recipes of Damocrates.’ See also *Antidotes* 1.5 (K14.32), 2.2 (K14.115)

54 See Fabricius (1972, p. 54), von Staden (1998, pp. 75–78), and Vogt (2009, p. 313).

55 See von Staden (1998, p. 76) and Vogt (2005, pp. 72–73).

56 Galen*, Antidotes* 1.7 (K14.44.16-18): ‘Since the elegiac verses of Andromachus are composed in a rather unclear manner (*asaphesteron*), it has seemed to me that the best thing would be to add the verses of Damocrates whose interpretation is entirely clear (*saphōs*).’ Galen does praise Andromachus for writing in verse, however: *Antidotes* 1.5 (K14.32). Andromachus’ poem is transmitted by Galen in *Antidotes* 1.6 (K14.32-42). Rendition in prose by Andromachus the Younger: *Antidotes* 1.7 (K14.42-44). Rendition into iambic trimeters by Damocrates: *Antidotes* 1.14 (K14.90-99). See Vogt (2005, pp. 63–73). On the poetry of Andromachus, see Heitsch (1964, pp. 8–15), Houston (1992), Luccioni (2003), and Hautala (2005).

57 See Vogt (2005, pp. 53–54) for a list of poets quoted by Galen.

58 See De Lacy (1966) and von Staden (1998).

59 On Galen and metaphors, see von Staden (1995b).

60 These works are listed in *On my own Books* 20 (173 Boudon-Millot, 2007b): ‘Ordinary words in Eupolis in 3 books; ordinary words in Aristophanes in 5 books; ordinary words in Cratinus in 2 books.’ See De Lacy (1966, p. 265) and von Staden (1997a, p. 52).

61 On didactic works in antiquity, see most recently Hutchinson (2009).

62 It should be noted that two short prose recipes follow these verse recipes. They may have been added at a later date.

63 I am referring to Lucretius’ *De rerum natura* 1.921-950 and 4.1-25.

64 *Types* 6.17 (K13.945.16-18)

65 *Types* 7.16 (K13.1058.5-10)

66 von Staden (1995a, 1997a) and Gleason (2009).

67 By the time he wrote his pharmacological books, Galen was far more reluctant to participate in public demonstrations: see *On my own Books* 3.14 (144 Boudon-Millot, 2007b).

68 Gleason (1995, p. 14). The orator impersonating the statue is Favorinus.

69 Gleason (1995, p. 158).

70 Thomas (1994, p. 415).
